# Thermography and Infrared Spectroscopy in the Detection of Periodontal Inflammation In Vivo: A Systematic Review

**DOI:** 10.3390/diagnostics16020222

**Published:** 2026-01-10

**Authors:** Heythem Nassim Guetatlia, Mickael Gette, Laurent Estrade, Victor Rimbaud, Frédéric Denis, Gaël Y. Rochefort, Matthieu Renaud

**Affiliations:** 1 N2Cox U1069 INSERM, Tours University, 37032 Tours, France; 2 Department of Medicine and Bucco-Dental Surgery, Tours University Hospital, 37044 Tours, France; 3 Faculty of Odontology, Tours University, 37032 Tours, France; 4 EA 75-05 Education, Ethique, Santé, Faculté de Médecine, Université François-Rabelais, 37000 Tours, France

**Keywords:** thermography, infrared spectroscopy, periodontal inflammation, non-ionizing imaging, periodontal diagnosis

## Abstract

**Background/Objectives:** Periodontal inflammation is a key feature of periodontal diseases, but traditional diagnostic methods are limited by invasiveness and radiation exposure. This systematic review aims to evaluate the potential of thermography and infrared spectroscopy for the in vivo detection of periodontal inflammation and to assess their reliability for clinical use. **Methods:** In accordance with PRISMA guidelines, an electronic search of the MEDLINE (PubMed) database was conducted to identify relevant studies published between 2000 and October 2025 that investigated these imaging modalities in periodontal inflammation diagnosis. **Results:** The search identified 310 records; after exclusions, 13 studies were included, comprising 7 thermography studies and 6 infrared spectroscopy studies, for a total of 712 patients. The included studies demonstrated the feasibility of thermography and infrared spectroscopy for detecting inflammatory changes in periodontal tissues in vivo. These non-invasive imaging techniques may help overcome the limitations of conventional clinical and radiographic diagnostic methods, particularly invasiveness and exposure to ionizing radiation. **Conclusions:** This field remains underexplored, and further studies are required to validate diagnostic performance, standardize methodologies, and determine their clinical applicability in routine periodontal practice.

## 1. Introduction

Periodontal disease is characterized by persistent inflammatory damage to the periodontal tissues [1,2]. Periodontal inflammation refers to the host’s immune response to microbial plaque, manifesting as redness, swelling, and bleeding of the gingival tissues, which can progress to tissue destruction in periodontitis. It is a key diagnostic criterion for periodontal diseases, including gingivitis (reversible inflammation confined to the gingiva) and periodontitis (irreversible loss of attachment and bone). It manifests as inflammation leading to the degradation and progressive destruction of the tooth-supporting structures, including gingiva, periodontal ligament, cementum, and alveolar bone [3]. Numerous studies also link it to the exacerbation of systemic conditions, such as cardiovascular diseases and diabetes [4,5,6]. The inflammatory process in periodontal disease is complex, involving a dysbiotic microbiome triggering a hyperinflammatory response, leading to tissue breakdown in periodontitis, unlike gingivitis, which is characterized by pathognomonic signs such as reversible gingival erythema, edema, and bleeding without attachment loss.

Diagnosis depends traditionally on clinical parameters, including periodontal pocket depth, bleeding on probing, and attachment loss [7]. These are supplemented by radiological assessments, which detect macroscopic signs of the pathology, such as alveolar bone resorption and loss of periodontal attachment [8,9]. However, early detection remains challenging due to the limitations of these methods. Limitations include the subjectivity and invasiveness of probing (causing patient discomfort and potential tissue trauma), delayed detection via radiography (only showing bone loss after 30–50% destruction), and radiation exposure. Emerging technologies like thermography and spectroscopy could improve early detection by non-invasively capturing subclinical inflammation through heat and hemodynamic changes.

The diagnostic methods for periodontal diseases in dental medicine have stagnated for years, prompting the exploration of alternative imaging modalities less ionizing and capable of providing an earlier diagnosis of periodontal inflammation, while ensuring their effectiveness in the daily practice of clinicians. Common tools include periodontal probes for pocket depth, bleeding on probing, and ionizing radiographs (e.g., periapical X-rays, CBCT) for bone assessment. Infrared technologies are considered due to their non-ionizing nature, reducing risks like cumulative radiation exposure, while providing real-time, contactless inflammation detection [10,11,12].

**Figure 1:**
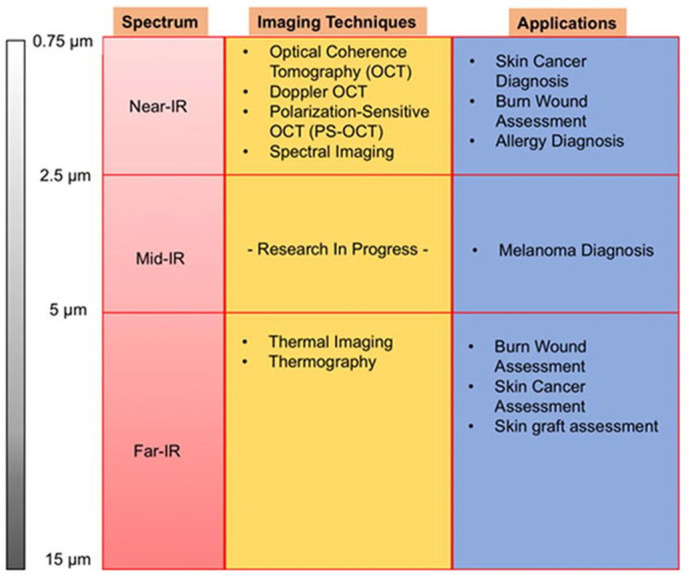
Summary of infrared imaging techniques across infrared spectra (adapted from [14]).

Infrared radiation was discovered by William Herschel (1738–1822) through experiments on “dark light,” extending Newton’s work on light diffraction. Herschel observed increasing temperatures beyond the visible red spectrum, revealing infrared radiation [13]. Unlike visible light, infrared wavelengths (0.7–1000 μm) are imperceptible to the human eye but ideal for analyzing tissue structure and dynamics. The infrared spectrum is divided into near-infrared (NIR; 0.75–2.5 μm), mid-infrared (MIR; 2.5–5 μm), and far-infrared (FIR; 5–15 μm) [14], each suited to specific imaging techniques (Figure 1). These technologies enable early visualization by detecting parameters like temperature gradients (in thermography, e.g., ΔT > 0.5 °C for inflammation) and tissue oxygenation levels (in spectroscopy, e.g., reduced oxyhemoglobin in inflamed sites), distinguishing from non-inflammatory processes like cancer, which may show different spectral signatures.

The two main analysis techniques for this spectrum of electromagnetic waves are thermography and spectroscopy:

Since the mid-1960s, multiple publications have introduced the use of infrared thermography as a potential non-ionizing tool for the detection of inflammation [15,16,17]. Far-infrared imaging, also known as infrared thermal imaging (IRT) or thermography, is a non-invasive, contactless imaging technique that provides real-time temperature measurements of the structures being analyzed by detecting their emitted far-infrared radiation (5–15 μm). This emitted thermal radiation is then converted into a visible and quantifiable infrared thermal image [18]. The measurement in this technique relies on detectors such as bolometers. These sensors receive the infrared emissions from objects, caused by the rise in their temperature. This thermal variation modifies the conductance of the sensor material, resulting in a change in the electrical signal measured at the output [14]. IRT has been rarely used in biomedical applications due to the limitations of early IR cameras in terms of performance and size. First-generation cameras provided insufficient thermal and spatial resolution, making the results unsatisfactory for many medical applications. Today, modern appliances with high sensitivity are used to produce high-resolution thermal images. As just seen, thermographic devices convert captured infrared radiation into electrical signals, which are then transformed into a thermogram visually representing temperature variations using a colormap [19]. The colors displayed on the generated images represent temperature gradients, ranging from the warmest shades, such as red, to the coolest ones, such as green and blue [18]. Some reviews have already discussed thermography in other fields, but studies dedicated to periodontology applications of this technique remain relatively rare. Up until now, no review has been conducted to highlight the use of these technologies in the diagnosis of periodontal inflammation.

Moreover, spectroscopy refers to the interaction between electromagnetic waves and analyses based on their wavelength. These interactions can be measured using various spectroscopic techniques that cover different wavelength ranges [20]. In the infrared region, spectroscopy can be used to analyze the spectra of near-infrared (NIR), mid-infrared (MIR), and far-infrared (FIR) [20,21,22,23]. Light penetration in imaging is strongly influenced by its interaction with chromophores, particularly hemoglobin and water. In the near-infrared (NIR) range (0.75–2.5 μm), water absorption remains low, allowing light to reach greater depths [14], making this spectral range a preferred alternative for this technique. Although many studies have demonstrated its effectiveness in other medical fields, the recent literature does not provide a comprehensive review of the current state of research and the prospects of IR spectroscopy in the diagnosis of periodontal inflammation, especially for its in vivo use.

Therefore, the aim of this systematic review was to explore the potential use of thermography and infrared spectroscopy in periodontology, with a particular focus on their in vivo application in the diagnosis of gingival inflammation.

## 2. Materials and Methods

This review was conducted following the principles established by the PRISMA guidelines [24]. The PRISMA 2020 checklist is provided in supplementary material. Despite promising applications in other fields, a knowledge gap exists in periodontology regarding their diagnostic performance (e.g., sensitivity/specificity), methodological standardization, and clinical readiness for routine in vivo use.

### 2.1. Search Strategy

The search covered publications available in the MEDLINE (PubMed) database over the past 25 years, aiming to identify studies published between 2000 and October 2025, analyzing the contribution of thermography and infrared spectroscopy in the diagnosis of periodontal inflammation. The following search terms and keywords were used, either alone or combined using the Boolean operators “AND”/“OR,” according to the following equation:

((infrared Spectroscopy) OR (near-infrared Spectroscopy)) AND ((Diagnosis) OR (Examination)) AND ((Periodontal inflammation) OR (periodontitis)) OR ((Thermography infrared) OR (Far-Infrared)) AND ((Diagnosis) OR (Examination)) AND ((periodontitis) OR (inflammation) OR (Periodontal inflammation)).

### 2.2. Study Detection

References of the eligible studies on the topic were manually checked, and two independent operators (N.G. and M.R.) screened the studies according to the inclusion/exclusion criteria. In case of disagreement, a 3rd reviewer (G.R.) was asked. No formal risk-of-bias tool was used due to study heterogeneity (e.g., varying designs, small samples); potential biases include selection (convenience sampling) and measurement (non-standardized protocols), addressed qualitatively. Quantitative synthesis/meta-analysis was not performed due to heterogeneity in designs, protocols, and outcomes (e.g., different temperature thresholds, spectral ranges).

#### Inclusion and Exclusion Criteria

The articles included in this review pertain to clinical studies conducted on human subjects with inflammation of the periodontal tissues. These studies focus on the diagnosis of inflammation in one or more periodontal tissues using real-time thermography or infrared spectroscopy, performed directly in a clinical setting on patients. Studies comparing inflamed to normal tissues were prioritized, with normal parameters defined as baseline temperature (e.g., 32–35 °C) or oxygenation levels (>60% oxyhemoglobin) from healthy controls.

The references of eligible studies on the topic were screened by abstract and full text, and studies were selected based on the inclusion/exclusion criteria.

Only studies concerning the use of the infrared subrange for periodontal inflammation detection were analyzed and included in this review, except for studies conducted on healthy periodontal tissues. These studies, which compare clinical and technical results in the evaluation of inflammation parameters, such as probing depth, were excluded in order to examine how infrared imaging can estimate these parameters and assess its potential for detecting inflammation.

Articles were excluded either directly from the title if they met an exclusion criterion, such as animal studies, or after reviewing the abstract or full text for the remaining articles.

The exclusion criteria were studies involving non-human subjects, those using techniques other than thermography or infrared spectroscopy, as well as articles addressing objectives unrelated to diagnosis (e.g., monitoring healing, evaluating therapy effects, or post-therapeutic outcomes), or diagnoses not related to periodontal tissues were excluded. Additionally, studies conducted outside the in vivo setting (ex vivo or in vitro) were also excluded.

Each study meeting the inclusion criteria was analyzed according to several parameters, including the study design, number of patients, age range, publication date, description of the camera or device used, as well as the objectives and findings.

## 3. Results

### 3.1. Study Selection

**Figure 2:**
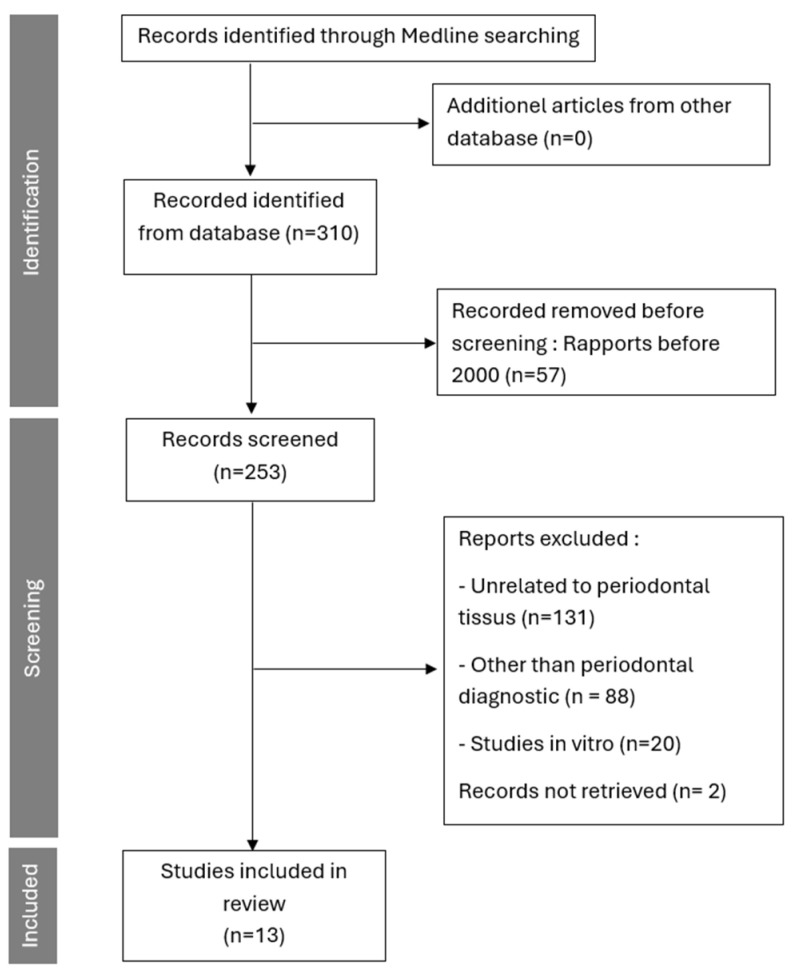
Study Selection Flowchart.

The search identified 310 published articles, 253 were published since the 1 January 2000. After applying the inclusion and exclusion criteria, 13 articles were included in this review (Figure 2). Among these articles, 7 focused on thermography, and 6 on spectroscopy as techniques for detecting periodontal inflammation, with a total of 712 patients.

#### 3.1.1. Presentation of IR Thermography Studies

Regarding thermography, among the 7 studies included in the analysis (Table 1), we were able to identify three common themes: room temperature, camera distance, and the spectrum used by the camera. Studies included in the analysis were clinical trials using thermography technique in comparison with standard clinical examination. Thermography studies consistently showed temperature increases of 0.5–2 °C in inflamed sites compared to controls, with methodological variability in camera distances (20–50 cm) and room temperatures (20–25 °C).

Regarding spectroscopy, among the 6 studies included in the analysis (Table 2), two common themes were highlighted: the biophysical properties used, and the spectrum range of the camera. The studies included in the analysis were clinical trials based on spectroscopy technique and compared to standard clinical examination. Spectroscopy studies demonstrated reduced tissue oxygenation (e.g., 10–20% lower in inflamed vs. normal sites) and elevated total hemoglobin, with consistencies in NIR range use but discrepancies in patient subgroups (e.g., higher variability in diabetics). Across thermography and spectroscopy, similarities include non-invasive detection of vascular changes, but discrepancies involve measurement depth and quantitative vs. qualitative outputs.

#### 3.1.2. Evolution of the Studies

**Figure 3:**
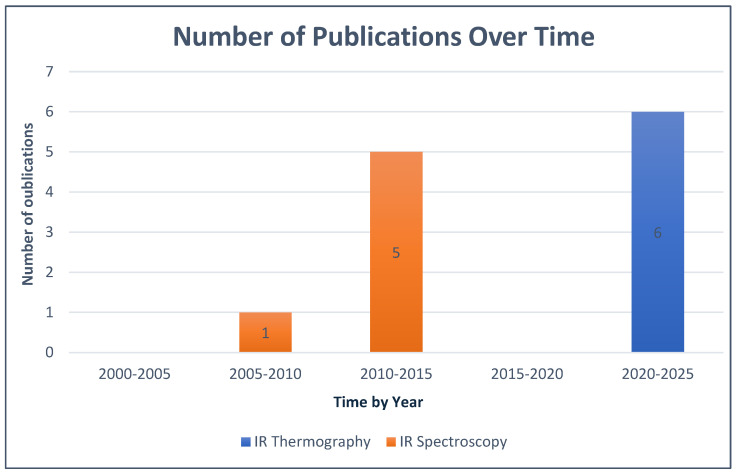
Evolution of Studies Over Time.

The application of IR spectroscopy and thermography for in vivo periodontal inflammation visualization was not widely practiced, and publications on this topic were limited. According to the publication periods used in this review, studies focusing on IR spectroscopy in this context only began in 2009, with no publications since 2015, while those on IR thermography in the same context only started later, in 2021. These data are presented below in Figure 3.

#### 3.1.3. Presentation of Cameras Used for IR Thermography

This section provides a brief overview of the most important aspects of infrared cameras used for periodontal inflammation detection through thermography.

The literature presents various camera models and their characteristics (thermal resolution, infrared sensor, acquisition frequency) collected directly from articles data or camera’s technical specifications available on the manufacturer’s website [38] (The data collected outside of the scientific articles from the PubMed database are indicated with an asterisk* in the table.) It is worth noting that all the articles included in the review mentioned the type of camera used.

The collected characteristics, along with the image analysis software, are presented in Table 3 below. The image analysis and processing software are listed for reference, with various software packages being used, including ThermaCam and Researcher Pro, which were employed twice in two different studies by the same team with two different versions.

#### 3.1.4. Overview of Devices Used for IR Spectroscopy

All spectroscopy studies utilized a miniaturized NIR device (e.g., Inspectra Spectrometer) with fiber-optic probes, measuring hemoglobin indices at 700–1000 nm, analyzed via custom software. All the studies included on infrared spectroscopy technology used the same diagnostic device because they were conducted by the same research team members, with the aim of validating the device on different subjects. The device and associated equipment used for performing the diagnosis and analyzing the results are presented in detail in Table 4 below.

## 4. Discussion

The host’s immune and inflammatory responses play a key role in the pathogenesis of periodontal disease. To formulate hypotheses and assess the feasibility of new periodontal diagnostic techniques, it is essential to first identify the clinical signs upon which we can rely to detect them in accordance with the technology used. In this review, two key signs were considered both reliable and potentially useful for improving periodontal diagnosis: edema and tissue temperature increase [39].

The heat emitted by tissues is a specific property detectable by infrared thermography [40]. In the context of periodontal inflammation, the increase in vascular dilation and permeability of the gingival tissues causes a higher heat production compared to normal state, which allows inflammation to be characterized using this technical criterion.

The included articles show that thermography can serve as an innovative diagnostic alternative or complement due to its non-invasive nature, particularly when radiological examination is contraindicated [25] such as in pregnant women during their first trimester. However, thermography has several limitations, such as limited tissue penetration [29], making it difficult to detect inflammation in the deeper tissues.

On a different note, IR spectroscopy primarily relies on edema to detect inflammation. Edema is a macroscopic sign resulting from the worsening of several microscopic processes related to hemodynamic changes during periodontal inflammation [37]. Among these changes, vascular dilation and increased permeability, as previously mentioned, are some of the most characteristic markers of gingival inflammation [35]. When infrared light passes through tissues, it selectively interacts with chromophores associated with oxygen, allowing for the real-time measurement of various tissue parameters like hemoglobin (oxygen carrier). This method offers a direct link to the oxygenation status and tissue perfusion. Most studies confirm that decreased tissue oxygenation is associated with an inflammatory state of the tissues, with a notable difference between periodontitis and gingivitis depending on the degree of inflammation. The more the inflammation progresses, the more tissue oxygenation decreases. In parallel, the tHB (total hemoglobin) index, which reflects regional blood volume, serves as an indicator of tissue perfusion status. Its elevation in periodontal areas, reflects altered vascularization, a characteristic of periodontal inflammation [32,33,34] as found in gingivitis or periodontitis.

Research teams have developed a miniaturized spectroscopy device suitable for periodontal use, based on near-infrared spectrum, the preferred choice for this technology [14]. This device has been validated by included studies investigating its applications on various subjects with different systemic conditions, such as diabetes, heart disease [32,33], or varying habits like smokers [34]. Overall, regarding infrared spectroscopy, the literature remains limited in its exploration beyond the use of a single device. This gap highlights the need for further research to expand its applications and compare them with other existing technologies for periodontal diagnosis. Compared to probing (invasive, subjective) and radiography (ionizing, late-stage detection), thermography offers real-time, non-contact heat mapping with high sensitivity for superficial inflammation, though limited penetration; spectroscopy provides quantitative hemodynamic data, superior for deeper assessment but requires device miniaturization. Advantages include safety and early detection, but challenges like cost and standardization persist. Thermography and spectroscopy share non-invasive feasibility but differ in depth (superficial vs. deeper); while technically viable, clinical applicability remains exploratory without validated thresholds, emphasizing need for larger trials over immediate use.

Because we focused on in vivo detection approach, the studies included in this review concentrate on periodontal inflammation tissue signs. However, this does not exclude the relevance for in vitro techniques, such as spectroscopy on saliva samples [41,42] or crevicular fluid [43,44], which remain potentially useful for early periodontal diagnosis.

In the same context, periodontal inflammation detection has been shown to be possible using near-infrared spectrum (near-infrared) through spectroscopy. However, there are other potential ways to exploit this light spectrum through similar innovative techniques, such as near-infrared Imaging (NIRI) technology, which is making its way into the oral healthcare field with the latest generation intraoral scanners [45,46]. Further studies are therefore needed to explore these new technologies through techniques already integrated into devices used in dental clinics.

Finally, to improve our review, it would be relevant to explore sources from other scientific disciplines, particularly databases used by biomedical imaging technicians and engineers, in order to obtain more detailed technical information on the technologies employed.

This review reveals the current literature on periodontal inflammation detection through the near and far IR spectrum. However, studies have shown the usefulness of other spectra on different tissues, such as skin tissue [14], Due to the histological similarity between mucosal and skin tissues, it is estimated that other spectra (mid-IR) and other techniques (Raman Spectroscopy) could be useful and exploitable for the detection of periodontal inflammation [43,47].

The use of medical imaging techniques in dental medicine is, to date, an underexplored field, which is why it represents an interesting research area.

## 5. Perspectives

The infrared spectrum, through these two approaches, shows promising potential that could contribute to improving the early diagnosis of periodontal diseases soon. However, further research is needed to identify the best practices for using this spectrum in this context.

With recent technological advancements in thermal cameras, infrared thermography is now a promising method for the detection of periodontal inflammation [30]. However, given these limitations, it is important to note that the clinical use of this technique still requires further in-depth studies to validate its effectiveness and improve its specific utility as an independent diagnostic tool.

Recent advancements, such as the integration of artificial intelligence with thermography, have shown enhanced accuracy in classifying gingival inflammation, particularly in specific populations like mouth breathers. This highlights the potential for AI to address limitations in manual analysis and improve diagnostic precision [31].

On the other hand, infrared spectroscopy, which has been used for years in the medical field, is making its way into periodontology through innovative devices designed by research teams [35,37]. This offers better prospects for reducing the size of spectrographs and facilitating their use by periodontists. This review highlights a promising start and the potential for exploiting this technique in the diagnosis of periodontal diseases, but there is still a long way to go before thermography becomes widely used in dental clinics.

Although no study has addressed this topic so far, a clinical study combining both techniques in a single device for periodontal diagnosis could be useful to maximize the effectiveness of these two techniques and achieve better diagnostic performance. This approach would allow one technique to offset the limitations of the other and/or simultaneously collect data from both techniques in order to achieve the best possible result through an expanded analysis of this data, facilitated by the introduction of artificial intelligence.

## 6. Conclusions

Numerous publications have demonstrated the potential of thermography and infrared spectroscopy (IR) for the in vivo detection of periodontal inflammation. These findings open new perspectives regarding the integration of these technologies into clinical periodontal practice in the near future. These non-invasive methods could allow a more precise and rapid assessment of the inflammatory status of periodontal tissues, providing a complementary tool to current diagnostic techniques. Although preliminary results are promising, this field remains largely underexplored, and several challenges must be addressed before widespread adoption. Further studies are needed to confirm the effectiveness, reliability, and reproducibility of these technologies in the daily practice of periodontists, considering the pathophysiological variables specific to each patient and the lack of standardized thresholds or validated metrics like sensitivity/specificity. These studies will help to determine the optimal conditions for their use and assess their impact on the early diagnosis of periodontal diseases in the future.

## Figures and Tables

**Table 1:** Overview of IR Thermography Studies.

References	Regions of Interest	Number of Patients	Room Temperature	Objectives	Camera Distance	Spectrum	Results
**Wziątek-Kuczmik D et al. 2024** [25]	Periapical areas of dead teeth	150	23 ± 1 (°C)	Examine the contribution of thermography in detecting asymptomatic infection sites in patients at high risk of systemic infections.	D = 0.4 ± 0.05 m	λ from 7.5 to 14 µm	The results showed a significant temperature difference between each patient group and the healthy group based on the measurement time.
**Bezerra de Melo N et al. 2023** [26]	Gums of the teeth	33	Between 20 and 25 °C (±1 °C)	Analysis of the clinical and thermographic parameters of gingival morphology to detect if there is an exploitable difference, as well as other parameters related to periodontal diagnosis.	D = 0.30 cm	λ from 7.5 to 14 µm	The results show a significant correlation between thermographic features and clinical gingival parameters.
**Wziątek-Kuczmik et al. 2022** [27]	Periapical regions of the teeth	8	23.0 ± 1 (°C)	Determination of the effectiveness of infrared thermal imaging for detecting the inflammatory response of periapical regions.	NC	λ from 7.5 to 14 µm	The results demonstrate a difference between the temperatures of the periapical regions of suspect teeth and those of the corresponding regions of healthy teeth, with an average temperature increase ranging from 0.51 to 0.39 °C.
**Derruau S et al. 2021** [28]	Left buccal space	2	NC	dentification of cellulitis in 2 patients through thermal imaging to validate its usefulness as a reliable diagnostic tool.	D = 0.4 ± 0.05 m	λ from 7.5 to 14 µm	The thermal results reveal a larger thermally activated area on the affected side compared to the healthy side, with a difference of more than 3 °C for Patient 1 and 2 °C for Patient 2.
**Delarue M et al. 2021** [29]	Periodontium of the posterior teeth of the mandibular arch	1	NC	Evaluation of tumor margins of small masses and/or tumors (peripheral ameloblastoma) not detected by conventional imaging.	NC	λ from 7.5 to 14 µm	The results indicate that the peripheral ameloblastoma region was hotter than the surrounding healthy tissues, with an increase in the thermal gradient from 1.5 to 2.5 °C.
**Aboushady et al. 2021** [30]	Periapical regions of the teeth	80	20.0 ± 1 (°C)	Evaluation of the validity of thermography for the diagnosis of periapical inflammatory lesions and the temperature ranges of acute pulpitis with apical periodontitis, acute and chronic periapical abscesses.	Indirect Tec: D < 2 m, Direct Tec: D = 20 cm	λ from 7.5 to 13 µm	The results demonstrate a significant difference between the average intra-oral thermal temperatures of the three diagnostic groups, with an increase in temperature in patients with acute pulpitis and apical periodontitis.
**Turgut Çankaya Z et al. 2025** [31]	Gingival tissues (thermal gingival images annotated and labeled based on bleeding on probing (BoP) and Gingival Index (GI) for inflammation severity)	40 participants (stratified by periodontal status and breathing pattern: mouth or nasal breathing)	Not specified (performed under standardized imaging conditions)	To detect and classify gingival inflammation severity using AI-supported analysis of thermal gingival images in patients with mouth breathing habits, and to establish specific thermal thresholds for gingival health and disease in this population	Not specified	Not specified (thermal imaging; typically far-infrared (FIR) in the 8–14 µm range for medical thermography, but no explicit details provided)	XGBoost classification achieved an accuracy of 92.74%, precision of 92.95%, sensitivity of 92.74%, and F1 score of 92.78%; cross-validation confirmed reliability with mean test score of 88.28% and validation score of 89.43%

**Table 2:** Overview of IR Spectroscopy Studies.

Ref.	Number of Patients	Average Age	Objectives	Key Parameters Measured	Spectrum	Results
**Duarte PM et al. 2015** [32]	78	Between 35 and 66 years	Evaluate optical spectroscopy as a periodontal diagnostic method for patients with type 2 diabetes and chronic periodontitis, while documenting the local hemodynamic profile at the periodontal level in these subjects.	The relative concentration of deoxygenated hemoglobin (Hb) and oxygenated hemoglobin (HbO2), the balance between oxygen supply and utilization in periodontal tissues.	λ from 0.5 to1.1 µm	In diabetic patients, tissue oxygen saturation and HbO2 levels were significantly reduced in periodontitis sites compared to gingivitis sites (*p* < 0.01). Furthermore, tissue oxygenation in healthy sites was markedly higher in controls than in diabetic subjects (*p* < 0.01).
**Zhang C et al. 2014 Maladie** [33]	121	Between 33 and 71 years	Verify the ability to identify periodontitis in patients with coronary artery disease using spectroscopy, with an instrument previously designed by the research team.			In coronary disease patients, a variation in Hb and HbO2 levels was observed (*p* < 0.01), and oxygen saturation was reduced in periodontitis sites compared to healthy sites in the diseased patients. In contrast, no difference in saturation was noted between the healthy groups and those with coronary artery disease.
**Liu KZ et al. 2014** [34]	54	Between 35 and 65 years	Analyze the effectiveness of spectroscopy in detecting periodontitis in smoking patients, using a device developed by the research team.			In smoking patients, tissue oxygen saturation significantly decreased in gingivitis sites (*p* = 0.016) and periodontitis sites (*p* = 0.007) compared to healthy sites. A trend of initial increase followed by a decrease in HbO2 concentration was observed, moving from healthy sites to affected sites.
**Ge Z et al. 2011** [35]	51	NC	Analysis of the hemodynamics of periodontal tissues during inflammation using optical spectroscopy.			The results reveal that tissue oxygenation significantly decreases between healthy sites, gingivitis sites, and periodontitis sites. This is explained by a notable increase in deoxyhemoglobin between healthy and gingivitis sites, as well as a significant decrease in oxyhemoglobin between gingivitis and periodontitis sites.
**Nogueira-Filho G et al. 2011** [36]	64	NC	Investigation of the diagnostic potential of optical spectroscopy in peri-implant inflammation in vivo.			The results indicate that tissue oxygenation at peri-implant sites was reduced compared to healthy sites (*p* < 0.05) due to an increase in deoxyhemoglobin and a decrease in oxyhemoglobin. Furthermore, the tissue hydration index, calculated from the optical spectra, was significantly higher in cases of mucositis compared to the other groups (*p* < 0.05).
**Liu KZ et al. 2009** [37]	30	Between 37 and 71 years	Analyze the ability of in vivo optical spectroscopy to simultaneously measure multiple inflammatory indices in periodontal tissues.	Tissue oxygenation, total tissue hemoglobin, deoxyhemoglobin, oxygenated hemoglobin, and tissue edema.		The results highlighted a decrease in oxygenation and an increase in deoxyhemoglobin at periodontitis sites, as well as a variation in the water index associated with electrolytes and temperature between the studied sites.

**Table 3:** Overview of Devices Used for IR Thermography.

References	Type of IR Camera	Image Resolution	Temperature Resolution	Acquisition Frequency	Image Analysis Software
**Wziątek-Kuczmik D et al. 2024** [25]	FLIR T1020	1024 × 768 pixels *	<0.02 °C *	f = 30 Hz *	ThermaCAM Researcher Pro 2.10
**Bezerra de Melo N et al. 2023** [26]	FLIR T650	640 × 480 pixels	0.05 °C	NC	FLIR Tools+™ 6.4
**Wziątek Kuczmik et al. 2022** [27]	FLIR T1020	1024 × 768 pixels *	<0.02 °C *	f = 30 Hz *	ThermaCAM Researcher Pro 2.8 SR-3
**Derruau S et al. 2021** [28]	VarioCAM^®^ HD	1024 × 768 pixels	NC	NC	IRBIS^®^ 3.1, InfraTec
**Delarue M et al. 2021** [29]	InfraTech VarioCAM HD	1024 × 768 pixels	0.03 °C	NC	NC
**Aboushady et al. 2021** [30]	FLIR E-5	120 × 90 pixels	NC	NC	FLIR Thermal Analysis and Reporting

**Table 4:** Overview of Devices Used for IR Spectroscopy.

References	Device	Probe	Power of the Light Source	Integration Time	Spectral Range	Resolution	Statistical Analysis Software
**Duarte PM et al. 2015** [32]	Portable spectro-graph PDA512-ISA	Custom bifurcated optical fiber probe for oral use	5 W	0.03 s	Between 500 and 1100 nm	5 nm	Statistica 7.1
**Zhang C et al. 2014** [33]							
**Liu KZ et al. 2014** [34]							
**Ge Z et al. 2011** [35]							
**Nogueira-F et al. 2011** [36]							
**Liu KZ et al. 2009** [37]							

## Data Availability

No new data were created or analyzed in this study. Data sharing is not applicable to this article.

## Supplementary Materials

The following supporting information can be downloaded at: https://www.mdpi.com/article/10.3390/diagnostics16020222/s1.
