# Supplementary material for: Thermography and Infrared Spectroscopy in the Detection of Periodontal Inflammation In Vivo: A Systematic Review
Source: Diagnostics (Basel). 2026 Jan 10;16(2):222. doi: 10.3390/diagnostics16020222 (PMC12840014; doi:10.3390/diagnostics16020222)
Supplement: Supplementary file 1 [file diagnostics-16-00222-s001.zip › diagnostics-4088746-supplementary.pdf]

Section/Topic	Item #	Checklist item	Location where item is reported
**Title**	1	Identify the report as a systematic review.	Title
**Abstract**	2	See the PRISMA 2020 for Abstracts checklist.	Abstract (see below for detailed Abstracts checklist)
**Introduction**			
**Rationale**	3	Describe the rationale for the review in the context of what is already known.	Introduction (paragraphs 1-3, discussing periodontal disease, limitations of traditional methods, and introduction to infrared technologies)
**Objectives**	4	Provide an explicit statement of the question(s) the review addresses.	Introduction (last paragraph: "the aim of this systematic review was to explore the potential use of thermography and infrared spectroscopy in periodontology, with a particular focus on their in vivo application in the diagnosis of gingival inflammation.")
**Methods**			
**Protocol and registration**	5	Indicate if a review protocol exists, if and where it can be accessed (e.g., web address), and, if available, provide registration information including registration number.	Not reported (only mentions following PRISMA guidelines, no protocol or registration mentioned)

Section/Topic	Item #	Checklist item	Location where item is reported
**Eligibility criteria**	6	Specify the inclusion and exclusion criteria for the review.	Materials and Methods, 2.2 Study Detection, Inclusion and Exclusion Criteria
**Information sources**	7	Specify all databases, registers, websites, programmes, etc., used to identify studies, and any contact with study authors, study sponsors, or trialists for obtaining unpublished data.	Materials and Methods, 2.1 Search Strategy (MEDLINE via PubMed; no mention of contacting authors)
**Search**	8	Present full electronic search strategy for at least one database, including any limits used, such that it could be repeated.	Materials and Methods, 2.1 Search Strategy (full search equation provided)
**Study selection**	9	Give the total number of studies screened; include a decision tree or flow diagram that shows the process of study selection.	Materials and Methods, 2.2 Study Detection (two reviewers, third for disagreement); Results, 3.1 Study Selection (numbers provided, Figure 2 flowchart)
**Data collection process**	10	Describe method of data extraction from reports (e.g., piloted forms, independently, in duplicate) and any processes for obtaining and confirming data from investigators.	Materials and Methods, 2.2 (analysis parameters listed; no explicit mention of extraction method or duplicate process, but independent screening implied)
**Data items**	11	List and define all outcomes for which data were sought, including prioritization of main and additional outcomes, and any methods used to classify presentation of findings.	Materials and Methods, 2.2 (parameters analyzed: study design, number of patients, age range, publication date, device description, objectives, findings)

Section/Topic	Item #	Checklist item	Location where item is reported
**Risk of bias in individual studies**	12	Describe methods used for assessing risk of bias in the included studies, including details of the tool(s) used, how many reviewers assessed each study and whether they worked independently, and if applicable, details of automation tools used in the process.	Not reported (added note in Methods about no formal risk-of-bias tool due to heterogeneity)
**Summary measures**	13	State the principal summary measures (e.g., risk ratio, difference in means).	Not applicable (narrative synthesis, no quantitative measures)
**Synthesis of results**	14	Describe the processes used to decide which studies were eligible for each synthesis (e.g., tabulating the study intervention characteristics and comparing against the inclusion criteria) (see item 13a).	Results (narrative, tables for overview)
	15	If done, describe any methods required to prepare the data for analysis (see item 13b).	Not applicable (no quantitative analysis)
	16	If done, describe any methods used to tabulate or visually display results of individual studies and syntheses (see item 13c).	Results (tables and figures used for display)
	17	If done, describe any methods used to synthesize results (see item 13d).	Methods (qualitative narrative synthesis due to heterogeneity)
	18	If done, describe any methods used to explore possible causes of heterogeneity among study results (see item 13e).	Not reported (heterogeneity mentioned but not explored quantitatively)

Section/Topic	Item #	Checklist item	Location where item is reported
	19	If done, describe any sensitivity analyses conducted to assess robustness of the synthesized results (see item 13f).	Not reported
**Reporting bias assessment**	20	Describe any methods used to assess risk of bias due to missing results in a synthesis (arising from reporting biases).	Not reported
**Certainty assessment**	21	Describe any methods used to assess certainty (or confidence) in the body of evidence for an outcome.	Not reported
**Results**			
**Study selection**	22	Give numbers of studies screened, assessed for eligibility and included in the review, with reasons for exclusions at each stage, ideally with a flow diagram.	Results, 3.1 Study Selection (310 records, 13 included, Figure 2)
**Study characteristics**	23	For each study, present characteristics for which data were extracted (e.g., study location, setting, dates conducted) and provide the citations.	Results, Tables 1 and 2 (overviews of studies)
**Risk of bias within studies**	24	Present assessments of risk of bias for each included study.	Not reported
**Results of individual studies**	25	For all outcomes considered (benefits or harms), present, for each study: (a) simple summary data for each intervention group (e.g., number of participants and events, mean and standard deviation for continuous	Results (narrative descriptions, tables with objectives and findings)

Section/Topic	Item #	Checklist item	Location where item is reported
		outcomes) and (b) effect estimates and confidence intervals, ideally with a forest plot.	
**Synthesis of results**	26	Present results of all statistical syntheses conducted. If meta-analysis was done, report the summary estimate and measures of uncertainty (e.g., confidence/credible interval). If comparing multiple interventions, report the ranking of the interventions (e.g., by the difference in mean outcomes, percentage ranking, or probability of a particular intervention being the best) and, if relevant, matrix of paired comparisons.	Results (narrative synthesis, no meta-analysis)
	27	Present results of all investigations of possible causes of heterogeneity among study results.	Not reported
	28	Present results of all sensitivity analyses conducted to assess the robustness of the synthesized results.	Not reported
**Certainty of evidence**	29	Present assessments of certainty (or confidence) in the body of evidence for each outcome from a well-performed review.	Not reported
**Discussion**			
**Summary of evidence**	30	Provide a general interpretation of the results and important implications.	Discussion (summary of findings)

Section/Topic	Item #	Checklist item	Location where item is reported
**Limitations**	31	Discuss any limitations of the evidence included in the review.	Discussion (limitations of techniques, literature gaps)
**Conclusions**	32	Provide a general interpretation of the results in the context of what is known, summarizing the conclusions and providing the implications for practice, policy or further research.	Conclusion
**Funding**			
**Other**	33	Describe sources of funding for the systematic review and other support (e.g., supply of data); role of funders.	Funding (no external funding)
**Competing interests**	34	Declare any competing interests of review authors.	Conflicts of Interest (none declared)
**Availability of data, code and other materials**	35	Provide a link to the complete dataset used for this systematic review, or alternatively one or more repositories or other secure data repositories where the complete dataset generated and analysed can be found (if not possible or appropriate, reasons should be explained).	Data Availability Statement (not applicable)
